# High-Resolution *In-Vivo* Analysis of Normal Brain Response to Cranial Irradiation

**DOI:** 10.1371/journal.pone.0038366

**Published:** 2012-06-04

**Authors:** Kelly Burrell, Richard P. Hill, Gelareh Zadeh

**Affiliations:** 1 Brain Tumor Research Centre, SickKids Research Institute, Toronto, Canada; 2 Ontario Cancer Institute/Princess Margaret Hospital and Campbell Family Institute for Cancer Research, University Health Network, Toronto, Canada; 3 University of Toronto, Toronto, Ontario, Canada; 4 Toronto Western Hospital University Health Network, Toronto, Canada; University of Frankfurt - University Hospital Frankfurt, Germany

## Abstract

Radiation therapy (RT) is a widely accepted treatment strategy for many central nervous system (CNS) pathologies. However, despite recognized therapeutic success, significant negative consequences are associated with cranial irradiation (CR), which manifests months to years post-RT. The pathophysiology and molecular alterations that culminate in the long-term detrimental effects of CR are poorly understood, though it is thought that endothelial injury plays a pivotal role in triggering cranial injury. We therefore explored the contribution of bone marrow derived cells (BMDCs) in their capacity to repair and contribute to neo-vascularization following CR. Using high-resolution *in vivo* optical imaging we have studied, at single-cell resolution, the spatio-temporal response of BMDCs in normal brain following CR. We demonstrate that BMDCs are recruited specifically to the site of CR, in a radiation dose and temporal-spatial manner. We establish that BMDCs do not form endothelial cells but rather they differentiate predominantly into inflammatory cells and microglia. Most notably we provide evidence that more than 50% of the microglia in the irradiated region of the brain are not resident microglia but recruited from the bone marrow following CR. These results have invaluable therapeutic implications as BMDCs may be a primary therapeutic target to block acute and long-term inflammatory response following CR. Identifying the critical steps involved in the sustained recruitment and differentiation of BMDCs into microglia at the site of CR can provide new insights into the mechanisms of injury following CR offering potential therapeutic strategies to counteract the long-term adverse effects of CR.

## Introduction

Radiation Therapy (RT) plays a pivotal role in the treatment of many CNS pathologies including CNS neoplasms, both primary infiltrative and metastatic brain tumors, and non-neoplastic disease processes, such as arterio-venous malformation [1], [2]. Unfortunately CR has significant adverse effects on the normal CNS, causing acute changes predominantly associated with CNS edema and debilitating cognitive decline, which manifest months to years after treatment. Paradoxically, patients who successfully survive their initial disease are left to face a progressive and severe decline in learning, memory and executive brain function [3]. Elucidating the precise molecular mechanisms that culminate in the adverse radiation effects following CR is crucial in preventing decline in function for future patients who require CR.

Postulated mechanisms of radiation-induced cranial injury include cyclic chronic inflammation, loss of oligodendrocytes and microvascular injury [4]. Recent research interest has focused on the reparative and therapeutic role that BMDCs can play in a number of CNS pathologies, including brain tumors, stroke, multiple-sclerosis and spinal cord injury [5], [6], [7], [8], [9], . The potential for BMDCs to play a similar reparative and therapeutic role in normal brain following CR has, so far, not been investigated, although Kioi *et. al.*, did demonstrate a significant contribution of BMDCs to brain tumor neo-vascularization [18]. Traditional histological methods are invaluable for tissue analysis however, they provide only ‘snap-shot’ cross-sectional information about the vasculature. Dynamic integration between different cell types and *in-vivo* tracking of cell migration is not possible with traditional cross-sectional analysis. Therefore, to complement histological analysis, we have taken advantage of 2Photon Laser Microscope (2PLM) high-resolution *in vivo* imaging of chimeric mice with fluorescent bone marrow chimeric mice. This experimental strategy has allowed us to visualize BMDCs at a single-cell level in normal brain and brain associated vasculature, longitudinally in real-time, to determine the spatio-temporal contribution of BMDC to neo-vascularization and in response to RT. Using this experimental strategy we demonstrate for the first time that there is a specific spatio-temporal and radiation dose-dependent recruitment of BMDCs that occurs following CR to normal brain. BMDCs persist, at the site of CR, long after the delivery of radiation, they migrate outside the vessel lumen and some encircle the vessel in part as smooth muscle cells, but do not form EC. Most notably our results establish that inflammatory progenitors are mobilized from the bone marrow, rather than being brain-resident inflammatory cells. This particular result provides invaluable therapeutic implications as BMDCs may be a primary therapeutic target to block acute and long-term inflammatory response following CR.

## Results

### BMDCs are recruited specifically to the site of CR

**Figure 1:**
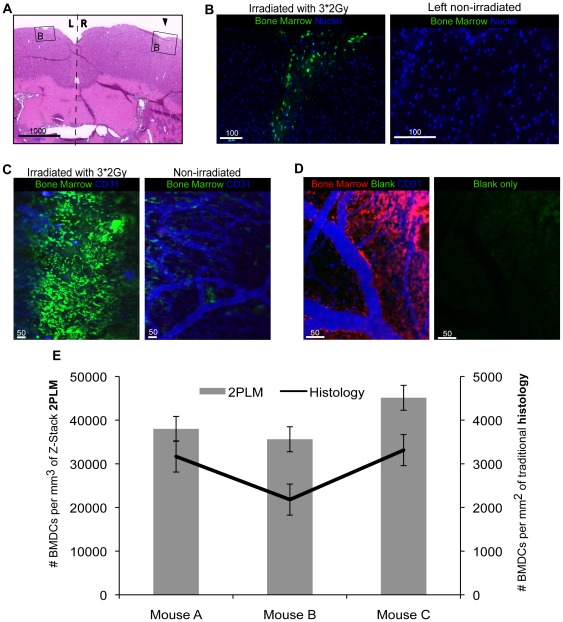
Bone Marrow Derived Cells Migrate Specifically to the Site of Cranial irradiation. (**A**) H&E staining of a normal brain 7 days following 3*2Gy radiation illustrates the location from where immunofluorescence images in (**B**) were obtained, 2× magnification. Black arrowhead shows the positioning of the intracranial window (ICW) and so the radiation path. (**B**) The right irradiated hemisphere demonstrates a trajectory of bone marrow derived cell (BMDC) recruitment which follows a cranial-caudal direction, with maximal recruitment at the cortex gradually decreasing beneath the cortical surface, compared to the contralateral-left, non-irradiated hemisphere which shows no BMDC recruitment (*Green: BMDC, blue: nuclei*), 10× magnification. (**C**) 2 photon laser microscopy *in-vivo* images 7 days post 3*2Gy radiation, illustrate distinct BMDC recruitment to the site of cranial radiation, whilst non-irradiated control brains demonstrate minimal incorporation of BMDCs (*Green: BMDC, red: blank, blue: CD31-APC*), 5× magnification. (**D**) To confirm that recruitment of BMDCs visualized on 2 photon laser microscopy is not as a consequence of green auto-fluorescent signals, red fluorescent chimeric mice were used to demonstrate a similar pattern of BMDC recruitment following 3*2Gy radiation but demonstrate a negative green channel (*Green: blank, red: BMDC, blue: CD31-APC*), 10× magnification. (**E**) Quantification of BMDCs visualized per field of tissue volume for 2PLM, blue bars, and traditional histology, black line. Note, the 10-fold difference in the scale bars highlighting the statistically significant difference in sensitivity of the 2 methods. This figure highlights the distinct advantage of 2 photon laser microscopy *in-vivo* imaging over traditional 5 µm cross-sectional histological analysis, clearly allowing visualization of higher levels of BMDCs in the same region of the brain, comparing (**B**) to (**C**) which when quantified in context of tissue volume (**E**) shows 2PLM is 10-fold more sensitive than traditional histology.

Using 2PLM imaging we observed a distinctive pattern of recruitment of BMDCs to the site of CR, as evidenced by the presence of Green Fluorescent Protein (GFP)^+^BMDCs as early as 1 hour post-RT, in a trajectory that parallels the radiation path in a cranial-caudal direction as seen with both immunofluorescence analysis and 2PLM imaging at the site of the Intra-Cranial Window (ICW) (Figure 1A,B,C). In order to confirm that the green fluorescent signal is not due to auto—fluorescence we used red fluorescent protein (RFP^+^BMDC) chimeric mice and see the same pattern of recruitment of BMDC to site of CR with no GFP^+^ signal evident (Figure 1D). The advantages of using a 2PLM *in-vivo* approach over traditional histological studies is significant, as illustrated by quantification of BMDCs recruited to the site of CR per volume of tissue (Figure 1E). There is a ten–fold increase in the number of detectable BMDCs in 2PLM, through the examination of multiple z-stacked images, when compared with traditional histology. Infiltration of GFP^+^BMDCs is very specific to the site of CR with minimal GFP^+^BMDCs present outside of the direct radiation beam. Furthermore, immunofluorescence examination of the contra-lateral non-irradiated hemispheres (Figure 1B), plus 2PLM images of control brains of non-irradiated mice (Figure 1C) showed minimal GFP^+^BMDC recruitment when compared to irradiated normal brain. To confirm that our observations were not dependant on mouse strain we demonstrate a similar pattern of recruitment in many strains of mice (ICR, Balb/C5 and NODSCID). We also saw the same pattern of recruitment in both our immediate (7 day post bone marrow transplant) and chronic (60+day post bone marrow transplant) chimeric models demonstrating no difference between BMDC recruitment between immediate circulating BMDC and complete reconstitution of BMDC. Furthermore, to verify that total body irradiation (TBI) does not bias our results of BM recruited to site of CR we used lead cranial shielding during TBI for BM reconstitution.

### Recruitment of BMDCs is radiation dose-dependent

**Figure 2:**
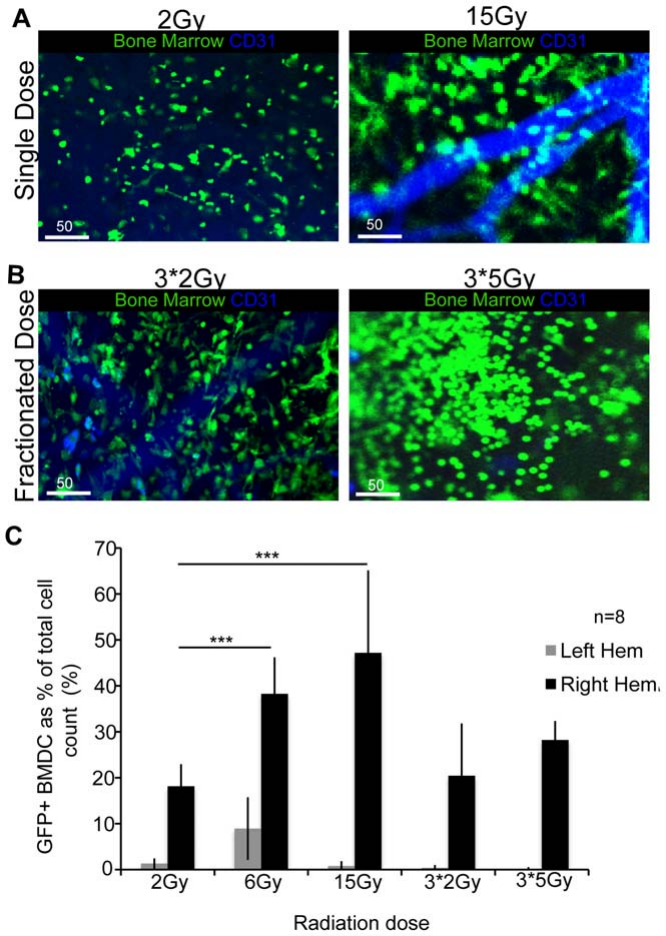
Bone Marrow Derived Cell Recruitment is Radiation Dose-Dependent. (**A,B**) Increasing levels of BMDC recruitment can be observed with increasing radiation doses on 2 photon laser microscopy *in-vivo* imaging 7 days post radiation (*Green: BMDC, blue: CD31-APC*), 10× magnification. (**A**) Single dose increase from 2Gy to 15Gy, (**B**) fractionated doses increase from 3*2Gy to 3*5Gy, all demonstrate an increase in BMDC recruitment. (**C**) Bar graph representing quantification of the extent of BMDCs recruited to site of cranial radiation 7 days post, demonstrating a statistically significant increase in BMDC recruitment between 2Gy to 6Gy and 2Gy to 15Gy, both p<0.0001***. Fractionated radiation demonstrates lower rate of BMDC recruitment when compared to its single fraction equivalent, perhaps explained by radiobiology principle of repair that occurs with fractionated radiation. The increase in BMDC recruitment is noted at all time points post radiation (1 hour, 1 day, 21 day).

Recruitment of BMDCs to the site of CR is radiation dose dependent. We delivered single doses of radiation, ranging from 2 to 15 Gy, plus two daily fractionated radiation regimens of 3×2Gy and 3×5Gy to the normal brain through the ICW. Using 2PLM imaging we demonstrated a visible increase in the extent of BMDC recruitment to the site of CR with radiation dose (Figure 2A,B), with the highest response seen at 15Gy at all time points post-RT. Quantifying the extent of BMDC recruitment demonstrates that with each higher radiation dose there is a statistically significant increase in BMDC recruitment (p<0.0001), with minimal BMDCs present on the contra-lateral left non-irradiated hemispheres (Figure 2C).

### Recruitment of BMDCs is time-dependent

**Figure 3:**
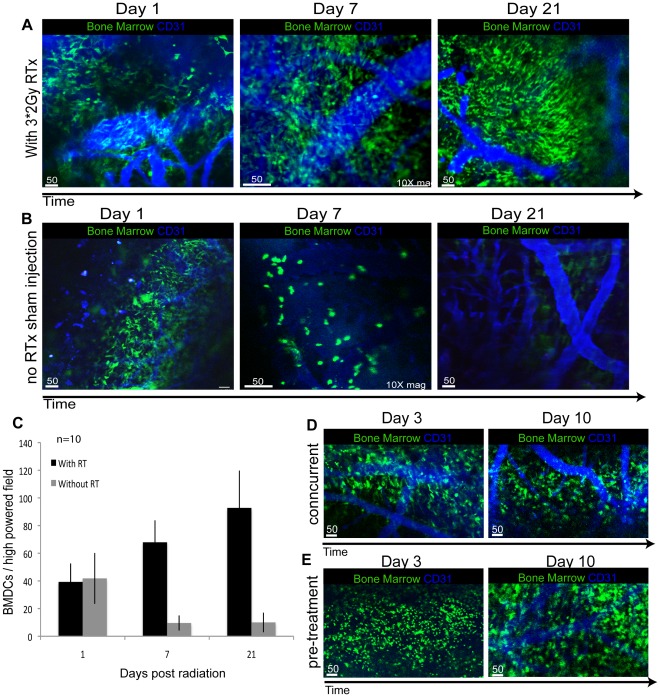
Longitudinal Pattern of Bone Marrow Derived Cells Recruitment to Cranial Irradiation. (**A,B**) The longitudinal fate and pattern of migration of BMDCs recruited to the site of cranial radiation is distinct from that seen in response to intracranial trauma. Using intra-vital EC staining antibody (CD31) it is clear that BMDCs do not contribute to vascular endothelial cells. (*Green: BMDC, blue: CD31-APC*), 5× magnification, unless stated. (**A**) BMDCs recruited to the site of 3*2Gy radiation in normal brain persist up to 1 month post radiation therapy, with a steady increase in extent of BMDCs present from day 1 up to day 21 as seen in the 2 photon laser microscopy images. BMDCs do not migrate or invade into the surrounding brain parenchyma with time, but stay within the radiation trajectory. (**B**) In control mice that received no radiation but received sham needle injection, there is evidence of BMDC recruitment to the site of injury within 24 hours of the sham injection but it rapidly diminishes before day 7 and is completely lost by day 21. (**C**) Quantification of the average BMDCs in high powered fields demonstrates the difference in patterns between RT and blunt traume. (**D,E**) AMD3100, an inhibitor of the Stromal-cell Derived Factor1/CXCR4 pathway, did not inhibit BMDC recruitment post radiation as shown in the 2 photon laser microscopy images (*Green: BMDC, blue: CD31-APC*), 5× magnification. (**D**) AMD3100 was administered concurrently with radiation and BMDC recruitment was unaffected from day 3 to day 10 post radiation. (**E**) Pretreatment with AMD3100 prior to radiation also did not show any significant difference in BMDC recruitment.

We monitored the recruitment pattern of BMDCs to the site of CR using 2PLM over a longitudinal period from 1 hour to 1 month post-RT. BMDC recruitment was seen as early as 1 hour post-RT, significantly increased by day 7 and then was maintained at a sustained level up to 1 month post-RT (Figure 3A). The recruited BMDCs persisted in the radiation field with no significant migration beyond the site of radiation. There was a distinctive and sustained presence of BMDCs at the site of CR well beyond the initial delivery of RT. This was not explained by proliferation of the initial population of BMDCs recruited to the site of CR, as established by lack of positive Ki67/BrDU staining of BMDCs *(data not shown)*. We compared recruitment of BMDCs in response to CR in normal brain with a blunt needle trauma to the normal brain. Blunt needle trauma in the frontal cortex resulted in early recruitment of BMDCs to the site of needle injury, however, the presence of BMDCs diminished rapidly after 7 days and was completely lost by 21 days (Figure 3B). Quantification of the BMDCs recruitment demonstrates the dramatic patterns seen in both models (Figure 3C)

### Investigating Mechanisms of BMDC recruitment: Stromal Cell Derived factor 1

We examined whether stromal-cell derived factor (SDF1)/CXCR4 signaling pathway was involved in the recruitment of BMDCs to the site of CR in normal brain. We treated animals with AMD3100 either two weeks prior to RT or concurrently with RT and examined alterations in the pattern of subsequent BMDC recruitment. We found no change in the extent of BMDC migration to the site of CR in normal brain following treatment with AMD3100, regardless of radiation dose or time course following RT (Figure 3D,E).

### Radiation induced microvascular alterations

**Figure 4:**
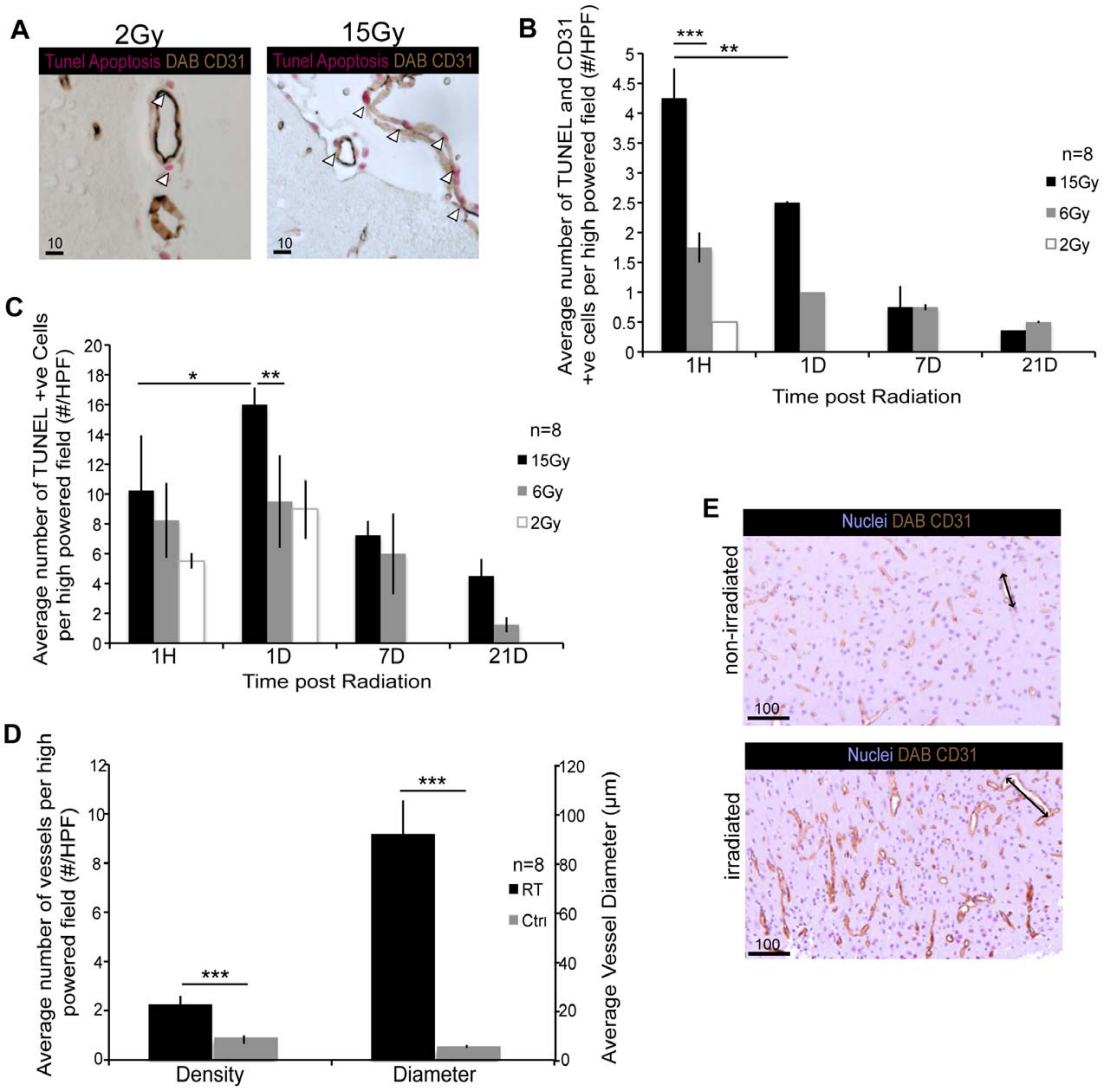
Microvascular Alterations Post Radiation. Immunohistochemical co-staining of CD31 and TUNEL confirms endothelial cell apoptosis occurs as early as 1 hour post radiation at site of cranial radiation. (**A**) Increasing radiation doses from 2Gy to 15Gy increases endothelial cell apoptosis as seen in immunohistochemistry sections *(Brown: CD31, Pink: TUNEL)*, 40× magnification. (**B**) Graphical representation of endothelial apoptosis, CD31^+^ TUNEL^+^, illustrates that 15Gy significantly induces the most endothelial apoptosis when compared to 2Gy and 6Gy at 1 hour post radiation, (p = 0.0001***) and that endothelial cell apoptosis levels at 15Gy 1 hour post radiation are significantly increased compared to 1 day post radiation, (p = 0.0004**). (**C**) Quantification of apoptosis of total parenchymal cells shows that 15Gy significantly induces most apoptosis (p = 0.0075**) at 1 day post radiation, however significantly more apoptosis is induced 1 day post radiation than at 1 hour post radiation (p = 0.0224*). Compared with what is seen with endothelial cell apoptosis it is clear that maximal endothelial cell apoptosis occurs earlier than overall parenchymal cell apoptosis. (**D**) Further analysis of vessels structure at the site of radiation, 7 days post 3*2Gy, demonstrates significant increases in both the density, p<0.0001***, and diameter of vessels, p<0.0001***, when compared to non-irradiated controls. (**E**) CD31 immunohistochemistry sections confirm the change in vessel density and diameter 7 days following 3*2Gy radiation when compared to non-irradiated control tissue, 10× magnification.

Microvascular injury, specifically endothelial cell (EC) apoptosis, is considered to play a pivotal role in radiation-induced cranial injury. We assessed the extent of EC apoptosis in a longitudinal and radiation dose dependent manner and in relation to the extent of BMDC recruitment, through double immunohistochemical staining for EC (CD31) and apoptosis (TUNEL) according to previous published protocols by Fuks *et a*l [21], [22]. A statistically significant (p<0.0001) increase in EC apoptosis (CD31^+^ TUNEL^+^) was observed with each increasing radiation dose, from 2Gy to 15Gy (Figure 4A,B). The highest degree of EC apoptosis occurred at 1 hour post-15Gy RT and diminished thereafter in a radiation dose and time dependent manner, decreasing significantly by 1 day post-RT, (p = 0.0001). At radiation doses less than 6Gy minimal EC apoptosis was observed. We also quantified overall apoptotic cells in the normal brain parenchyma (TUNEL^+^). The peak of apoptosis in the normal brain parenchyma occurred significantly later than EC specific apoptosis, with the highest level seen 1 day post-RT (p = 0.0224). The highest degree of parenchymal TUNEL^+^ cells was seen in response to 15Gy, which was significantly higher than that seen with the other radiation doses (p = 0.0075) (Figure 4C). We analyzed alterations to vascular structure in response to CR by measuring microvascular density, vessel diameter and vessel leakiness. There was a statistically significant increase in vessel diameter by 7 days post-RT together with an increase in microvascular density (p<0.0001) (Figure 4D,E). Increased vessel leakiness was detected at 7 days following CR using Evans Blue perfusion fixation (Figure S1A). We investigated whether there was an associated change in cerebral blood flow (CBF) with ultrastructural changes observed in the vasculature at the site of CR. We used MRI flow-alternating-inversion-recovery techniques to measure CBF, comparing irradiated (R) hemisphere to non-irradiated (L) hemisphere. We found that at site of CR associated with the area of increased MVD and dilated vessels there was no significant change in CBF (Figure S2).

### Contribution of BMDCs to vascular elements following cranial irradiation

**Figure 5:**
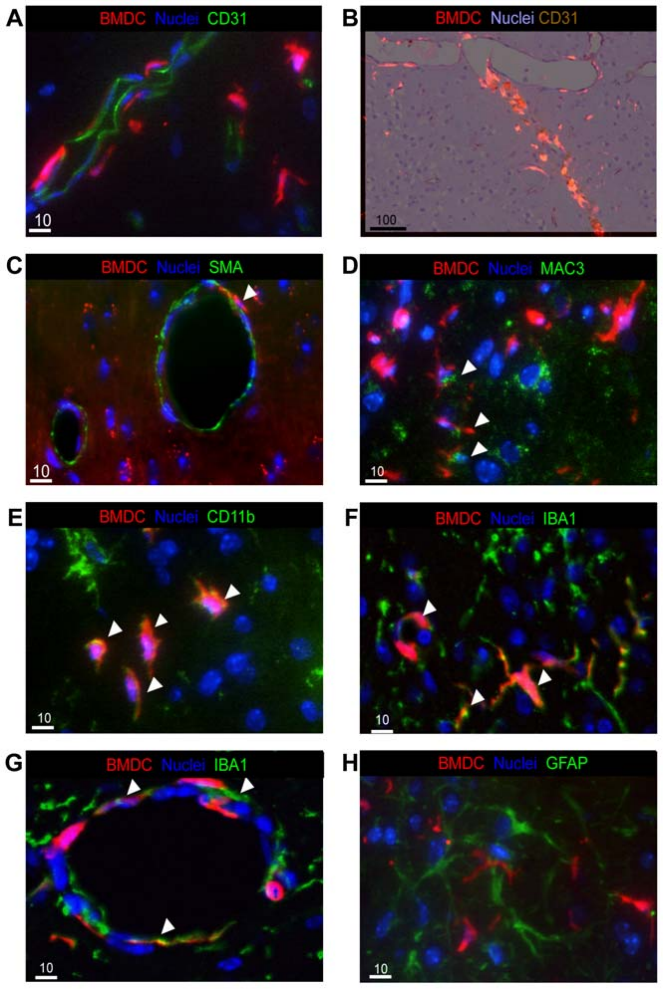
Characterization of Bone Marrow Derived Cells. (**A–H**) Immunofluorescence analysis of brain sections for characterization of cell types that BMDCs potentially differentiate to at the site of cranial radiation 7 days post 6Gy radiation, in the staining a red fluorescent chimeric mouse was used. (*Green: stain, Red: BMDC, Blue: nuclei*), 40× magnification (**A,B**) No differentiation of BMDCs to vessel endothelial cells is evident, confirming what was seen on the 2 photon laser microscopy *in-vivo* imaging. (**A**) BMDCs do not co-localize with the endothelial cell marker CD31, using confocal immunofluoroscence, or (**B**) immunohistochemical overlay of CD31 (*Brown: CD31*), 10× magnification. (**C**) Few BMDCs co-localize with the pericytic differentiation marker smooth muscle actin, shown by white arrowhead. (**D,E**) Approximately 40% of recruited BMDCs stain positively for inflammatory markers: (**D**) MAC3, co-localization shown by white arrowhead, (**E**) CD11b, co-localization shown by white arrowhead. (**F**) 50% of recruited BMDCs found in the parenchyma co-localize with the microglial differentiation marker IBA1 shown here by white arrowhead. (**G**) IBA1^+^ BMDCs are also found in the perivascular space around vessel wall, shown by white arrowhead. (**H**) There was no astrocytic differentiation or glial scaring, as determined by the lack of GFAP co-localization.

We took advantage of our 2PLM *in-vivo* intra-vital imaging strategy to examine *in-vivo* differentiation of BMDCs intracranially following CR, with specific interest in whether BMDC differentiate to form EC. We used intravenous injection of CD31^+^ tagged Allophycocyanin (APC) antibody to allow specific *in-vivo* identification of vessel ECs. Additionally, the use of time-lapse footage (Video S1) and z-stack imaging (Video S2) provided detailed information on three-dimensional correlation of BMDCs with the vasculature and in depth analysis of their relationship to the vessel wall and vessel ECs (Video S2 & Figure S3). BMDCs did not differentiate to form EC at any point following CR or at any radiation dose. On 2PLM images we saw BMDC migrate to the site of CR, migrate outside of the vessel lumen, where a subpopulation went onto encircle the vessel, but did not form CD31^+^APC cells. Migration of BMDCs out of the vessel lumen was independent of vessel size, caliber or location along the course of the vessel. There was no difference between branching points in comparison to regions along the vessels between branching points. This is in contrast to what is reported for cancer cell metastases by Winkler *et al* where a majority of metastatic cancer cells migrate out of the tumor vasculature at sites of branching [23]. Immunofluorescence and immunohistochemical analysis both at site of CR and outside the radiation field did not show any co-localization of CD31^+^ cells with BMDCs (Figure 5A,B). Immunofluorescence staining for smooth muscle actin (SMA) confirmed a small, although not statistically significant, increase in the extent of SMA^+^ BMDC that surrounded the vasculature in the area of CR, compared to the contralateral control non-irradiated brain. However, the majority of peri-vascular BMDCs are not SMA^+^ (Figure 5C) rather they are inflammatory and microglial-like cells as described below.

### BMDCs differentiate to form specific cell types, beyond vascular elements

We used immunohistochemical analysis to examine the cell types into which recruited BMDC differentiate using a panel of antibodies (CD31, SMA, MAC3, CD11b. CD11c, F4/80, IBA1, GFAP) for inflammatory, immune, vascular and brain cells. Of all cell types explored two cell population predominated: inflammatory cells and microglia. Using a panel of pro- and anti-inflammatory markers; Mac3, CD11b, CD11c, F4/80, we demonstrated that approximately 40% of the recruited BMDCs in the brain are inflammatory cells (Figure 5D,E). At the site of CR most, if not all, of the inflammatory markers co-localize with BMDC, suggesting recruited progenitors from the bone marrow and not brain resident inflammatory cell populations are responsible for initiation of the inflammatory response seen post-RT.

The second significant cell type that BMDCs differentiated into was microglia. BMDCs recruited to the site of CR formed microglia or perhaps more accurately microglia-like cells in a radiation dose dependent manner. These bone marrow derived microglia were seen in addition to resident microglia in the brain parenchyma and in peri-vascular regions (Figure 5F,G). Above doses of 6Gy, approximately 50% of the detectable microglia were bone marrow-derived, with the other half being resident microglia that showed no co-localization with BMDCs (Figure S1B). Minimal levels of bone marrow derived microglia were seen outside the site of CR or in the non-irradiated contra-lateral hemispheres of the mice.

## Discussion

Cranial irradiation (CR) plays an invaluable role in the treatment of various CNS pathologies [3], [24], including all primary and metastatic brain tumors and other non-oncological processes such as vascular malformations of the brain. Despite the recognized therapeutic efficacy of CR there are significant negative consequences on normal brain. Little is understood about the molecular mechanisms responsible for the adverse effects following CR. In this study we have shown for the first time using *in-vivo* high-resolution imaging, that BMDCs are recruited to the site of CR in a highly distinct temporal-spatial and radiation dose dependent manner (Figure 1,2). Taking advantage of the high resolution of optical imaging we have been able to confirm a distinctive pattern of BMDC response that has previously not been detected with use of routine histological analysis. BMDCs were seen specifically at the site of CR and minimally outside of the radiation field or in non-irradiated brains. There was a statistically significant increase in the extent of BMDCs recruited to the site of CR with increasing radiation dose. These findings are a critical first step in understanding the involvement of BMDCs in response to CR.

Disruption of the blood-brain barrier (BBB) is a consistent and recognized event following radiation injury to the brain, resulting in increased vascular permeability and leakiness of vessels [25], [26]. We observed dilated vessels with increased leakiness at the site of CR as demonstrated by Evans blue extravasation from the intracranial vasculature at site of CR (Figure S1A). Thus presence of BMDCs at the site of CR might be explained by direct influx of BMDCs from intracranial vessels that have lost their BBB integrity. Previous data has reported that lymphocytes are recruited to the site of RT injury and can attach to vessel EC and infiltrate into normal brain parenchyma [27], [28]. We examined this possibility by staining for possible peripheral blood cells such as erythrocytes, granulocytes, megakaryocytes, Natural Killer cells, T and B cells using immunohistochemistry and immunofluorescence analysis in addition to 2PLM imaging. BMDCs recruited to the site of CR did not stain positively for the lymphocytes markers; CD3 and B220, and very few BMDCs showed characteristics of red blood cells at the site of CR implying that BMDCs present at the site of CR do not simply represent an influx of peripheral blood through disrupted BBB of intracranial vessels. Another explanation is that BMDCs contribute to long-term glial scarring or fibrosis seen post-RT. This postulate was tested using GFAP (Figure 5H) and reticulin stains and no co-localization of BMDC was observed.

There was sustained BMDC recruitment long after the initial delivery of RT to the normal brain, which was not explained by proliferation of the initial population of BMDC recruited to site of CR. The fact that BMDCs persist specifically at the site of CR implies a continued level of injury, possibly reflecting the process of apoptosis of ECs or other cells, such as oligodendrocytes, which in turn would release cytokines and chemokines [24] that trigger BMDC recruitment. SDF1/CXCR4 is the signaling pathway most implicated in regulating recruitment and migration of BMDCs in tumor models [18], [20], [29]. AMD3100 is a known inhibitor of the SDF1 pathway and has been shown to cross the BBB to diminish or obliterate BMDC recruitment in intracranial tumor models. We explored the possibility that SDF1/CXCR4 mediated the recruitment of BMDCs in response to CR in normal brain. However, we found that AMD3100 did not block recruitment of BMDC to site of CR, regardless of radiation dose or time course following RT (Figure 3C,D). There is evidence to suggest that SDF1/CXCR4 signaling of BMDCs is highly dependent on the microenvironment [18], [20], [29] and based on our results SDF1/CXCR4 does not appear to play a significant role in recruitment of BMDC in the context of CR in normal brain. These initial studies are an attempt to investigate the mechanism responsible for BMDC recruitment, but further studies are required to fully elucidate the precise signaling pathways involved since the most recognized pathway, SDF1, does not appear to be involved following RT.

Considerable current research is focused on the contribution of BMDC to *de novo* vessel formation or the process of vasculogenesis in response to insults such as ischemia or tumor growth [20], [30], [31], [32], [33], [34], [35]. Most relevant, is the recent eloquent study by Kioi *et.al.* which demonstrated that vasculogenesis is relevant to tumor reoccurrence and progression following RT [18]. Given the central role that microvascular injury plays in triggering radiation induced injury [21], [28], [36], [37], [38], [39], [40], [41], we examined the potential role that BMDCs play in repairing injured microvasculature in normal brain. In particular, we focused on whether BMDCs differentiated into the full complement of cells necessary to compose a functional vessel to aid the process of vasculogenesis and directly replace apoptotic EC or, more indirectly, whether BMDCs form peri-vascular support cells to help in the repair of injured microvasculature.

The extent of EC apoptosis following CR remains controversial, as results vary between studies [25], [39], [42], [43]. Therefore, we first established extent and timing of EC apoptosis in relation to BMDC recruitment in our experimental model. Endothelial cell apoptosis precedes brain parenchymal cell apoptosis by approximately 24 hrs (Figure 4B). Though EC apoptosis precedes BMDC recruitment, BMDC recruitment is sustained well after EC apoptosis (beyond 7 days), supporting the possibility that released chemokines and cytokines continue to maintain memory of RT injury. In addition to EC apoptosis, alterations in vascular architecture and microvascular density are evident as a consequence of CR. There is a statistically significant increase in vessel diameter by 7 days post-RT that persisted beyond 1 month without repair (Figure 4D,E). It appears that the timing of BMDC recruitment follows a peak in EC apoptosis in the CNS. These results are in keeping with previous published data that suggests a pivotal role for EC apoptosis triggering a cascade of molecular events that culminates in cellular injury in the CNS. Following CR, recruited BMDCs migrate outside of the vessel lumen, where a subpopulation remain intimately involved with the vasculature and encircle the vessel lumen. At no stage post-RT did we observe recruited BMDCs to form EC regardless of radiation dose. A percentage of BMDCs surrounding the blood vessels exhibit smooth muscle cell characteristic, however the majority of peri-vascular BMDCs are not SMA^+^ (Figure 5C). This can be explained by differential stages of smooth muscle cell maturity and differentiation or that BMDCs which encircle the vessel lumen provide a supportive role that is distinct from that provided by normal SMC. Our observation that BMDCs do not replace apoptotic EC following CR in normal brain is in contrast to results from the recent study by Kioi *et.al.* and other groups that showed distinct differentiation of BMDCs to form ECs following RT in glioma models [18], [19], [20], [44], [45], [46], [47]. Taken together, prior studies have predominantly focused on the contribution of BMDC in tumor models and have suggested that possibly an oncogenic signal may be necessary to promote and drive BMDCs differentiation to an EC fate and contribute to vasculogenesis. However, in the context of CR in normal brain, and perhaps in the absence of an oncogenic signal, we do not see any differentiation of BMDCs to form EC.

Acute and chronic cyclic inflammations are postulated mechanisms involved in radiation induced cranial injury, however, the origins of the inflammatory response has never been established [48], [49], [50]. Our results confirm, for the first time, that the inflammatory response seen in the brain post-RT is largely mediated through mobilization of bone marrow derived inflammatory progenitors and not by inflammatory cells resident in the brain, demonstrated by the lack of inflammatory cells that are not GFP^+^BMDC at site of CR (Figure 4D,E). The peri-vascular localization of some of the inflammatory BMDCs suggests they may play a role in the initial steps of repair of apoptotic and damaged vessel endothelium.

A critical finding in this study is the differentiation of BMDCs to form microglia. Microglia are recognized as a unique macrophage population within the CNS, and are considered both as cells of neuroepithelial origin and as mononuclear phagocytes involved in inflammatory and immune responses [51]. They play a crucial role in neuronal development during embroygenesis [52], [53], [54], [55] and during adult life their principle role is thought to be protective, either through an anti-inflammatory role, removing debris and dead tissue in an attempt to restore normal tissue function [56] or via release of neurotrophic factors that have essential roles in neuroprotection and repair [57]. There is significant controversy surrounding the origins of microglia, highlighted by the recent community corner review in *Nature Medicine 2010* [58]. Though microglia in the developing CNS are thought to be derived from myeloid-monocytic lineage cells, in the adult brain the BBB heavily regulates microglia, and so the origins of adult microglia in the CNS and how they are replenished remains disputed [56]. Most current literature suggest that peripheral myeloid cells from fetal and adult hematopoiesis contribute minimally if at all to adult microglia in the CNS [58]. Previous studies in irradiation chimera models show only a 5% engraftment of microglia from reconstituted bone marrow [59] but no studies have focused specifically on response of microglia and its origins in CR. Mildner *et. al*, demonstrated recruitment of monocytes to demylinating brain from bone marrow and observed that they differentiated into microglia [60]. Here we demonstrated that in irradiated brain BMDCs are recruited and in a dose dependent manner constitute up to 50% of the microglia at the site of CR but there is minimal contribution of BMDCs to microglia outside of the radiation field.

The plasticity and tremendous potential for differentiation and transformation of BMDC into cells relevant to the microenvironment and disease site has been shown previously by a number of authors [60], [61]. Most relevant to this study, Piller *et. al.* demonstrate differentiation of BMDC into neuronal phenotypes, specifically Purkinje neurons, 1–6 months following bone marrow transplantation [61]. More recently Nern *et. al.* demonstrate a transient phenomenon which argues that in fact BMDCs fuse with resident purkinje cells rather than transforming into them [62]. Our data is in support of the ability for BMDC to differentiate into microglia or microglia-like cells in response to the microenvironmental requirement of irradiated normal brain.

Our key novel findings include a specific temporal-spatial and radiation dose dependent recruitment of BMDCs that occurs following CR to normal brain. BMDCs persist long after the initial insult of radiation injury and remain at the site of CR with minimal migration outside the radiation field. BMDCs migrate outside the vessel lumen to encircle the vessel in part as smooth muscle cells, but predominantly as inflammatory cells and microglia, to possibly provide a vascular support or reparative role. BMDCs do not form EC or regulate *de novo* vessel formation or vasculogenesis at any radiation dose or time course following CR.

Our results identify that inflammatory progenitors mobilized from the bone marrow, versus those brain resident inflammatory cells, are the primary source of the acute and chronic inflammatory response that is considered to trigger the onset and progression of neural injury seen post-RT. This particular result provides invaluable therapeutic implication as BMDCs may be the primary therapeutic target to block acute and long-term inflammatory response following CR. Most notably we provide evidence that more than 50% of the microglia are not resident microglia but are recruited from the bone marrow following CR. In disease processes such as Alzheimer's bone marrow derived microglia have a more efficient therapeutic potential compared with resident microglia in eliminating amyloid plaques [63], [64]. Therefore, it is highly relevant to explore the therapeutic potential of microglia derived from BMDCs in response to CR in order to diminish or counteract the adverse effects seen following CR.

## Methods

For all experiments, a total of 15 chimeric NODSCID mice were generated for each condition. This allowed five mice per time point to be imaged using the 2PLM and two mice to be sacrificed for histological analysis and additional mice accounted for animal loss. All experiments, CR, needle trauma and control were repeated in triplicate. We used both immediate (7 day post BMT) and chronic (60+day post BMT) chimeric models. We examined the brains using 2PLM and histological analysis at 1 hr, 24 hr, 3day, 7day, 10day, 14day and 21day post radiation and needle trauma (Figure S4).

### Bone Marrow Reconstitution

NOD/SCID, ICR background and Balb/C5 chimeric mouse models were created to have GFP+ or dsRED (RFP)+ bone marrow through bone marrow reconstitution [29], [33]. Briefly, donor transgenic mice constitutively expressing green or red fluorescent protein (GFP/RFP) were sacrificed according to institutional guidelines and bone marrow was harvested from both tibias and femurs into 1 ml of sterile saline (VWR). Eight-week old recipient mice were lethally irradiated with 3Gy total body irradiation using a Gamma Cell 40 irradiator (Nordion International). To ensure results were not biased by exposing the brain to irradiation cranial shielding was used. 6×10^6^ donor-derived bone marrow cells were injected intravenously via the dorsal tail vein. Efficient reconstitution was confirmed by post-sacrifice examination of bone marrow and circulating blood for GFP^+^ or RFP^+^cells for every study time point up to six months to confirm complete BMDC engraftment, with approximately 70% engraftment seen. For every experimental mouse BM and blood was collected to ensure equal percentage of BM engraftment at time of necropsy (Figure S5).

### Generation of Intracranial Window

Mice were anaesthetized using 0.5 mg/g of Avertin (Sigma Aldrich) and 5 mg/kg of pre-surgical analgesic Caprofen©. TearGel (Novartis) was applied to prevent corneal dehydration. A mid-line scalp incision was made from the ears to the eyes to expose the skull and the underlying periosteium was frozen with 2% Lidocaine∶Epinephrine (Bimeda MTC) before dissecting away. A high-speed drill with a 2.7 mm trephine (Fine Science Tools) was used to generate a circular bone flap in the right frontal skull. The cortical surface was kept hydrated using saline and a 5 mm glass coverslip (Warner Instruments) was placed over the window. The skull surface was dried and self-curing dental acrylic (Bosworth) was used to seal the coverslip onto the skull surface.

### Drug therapy

AMD3100 resuspended in saline was administered to mice 1.25 mg/kg twice daily subcutaneously for a continuous two-week period either beginning with RT or given 2 weeks prior to RT.

### Stereotactic Radiation Therapy

Mice were anaesthetized using isofluorane at 4% for induction followed by 1.5–2% throughout the procedure with 0.5–1 liter O_2_ a min. Mice were placed on a custom-made frame designed to immobilize the head, and the frame placed inside a micro-irradiator (Precision Xrad 225Cx) set up in-house for *in-vivo* targeted RT research. A 360° Computer Tomography scan of the mouse inside the Xrad was taken with the Xray tube running at 40 kVp and 0.05 mA through a 2 mm Aluminium filter, and was used to adjust the platform to centre the RT beam to the right hemisphere, specifically the ICW. RT was administered through a 8 mm×11 mm collimator, with the Xray tube running at 225 kVp and 13 mA, through a 0.93 mm Copper (Cu) filter, from the top (dorsal) and from the bottom (ventral) of the gantry (Figure S6).

### Optical Imaging

At specified time points (1 hr, 24 hr, 3 dy, 7 dy, 10 dy, 14 dy, 21 dy) mice were anaesthetized using 0.5 mg/g of Avertin (Sigma Aldrich). Ten minutes prior to imaging Allophycocyanin (APC) tagged CD31 antibody (BDPharmingen), 0.2 µg/g, was intravenously injected. The mouse was inverted onto custom-built restrainer, designed to stabilize head perpendicular to the laser, and mounted onto 2PLM-automated stage. GFP, RFP and APC were excited with 488 nm, 543 nm, 633 nm wavelengths respectively and emissions collected using the BP500-550IR, BP565-615IR, BP650-710IR filter sets respectively using a line step of 1 and averaging of 4. Time elapsed videos were made using a line step of 8 and average of 1, to highlight movement of the BMDCs in the field, for 150 msec continual loops (Video S1). Z-Stack images were acquired using 5 µm intervals over 50–100 µm depth of field with reduced averaging of 2 to reduce imaging time (Video S2).

### Measuring Cerebral Blood Flow using Magnetic Resonance Imaging (MRI)

MR imaging was performed with a 7 Tesla Biospec 70/30 (Bruker Corporation, Ettlingen, DE), using the B-GA12 gradient coil insert and 7.2 cm inner diameter linearly polarized volume resonator coil for Radio Frequency (RF) transmission. Mice were oriented in supine position on a slider bed and maintained at 1.8% isoflurane. A dedicated murine head coil was used for RF reception. Neuroperfusion (P) was quantified in a single transverse imaging slice, predefined to highlight the hippocampal region, using a flow-alternating-inversion-recovery (FAIR) technique, which quantifies the difference in T1 relaxation rate between global and slice-selective inversion pulses, as follows:
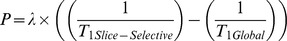
Equation 1 - Where P is Neuroperfusion (ml blood/(100 g tissue*min) and lambda is the blood-brain partition coefficient for water (90 ml/100 g) as previously described [65], [66], [67].

Each T1 measurement (selective and global) was quantified from MR signals at 18 inversion recovery times ranging from 25 ms to 6825 ms, in 400 ms increments. Each image was acquired with a single-shot echo-planar imaging readout (14.5 ms echo time, 221 kHz readout bandwidth) with a slice thickness of 1 mm. 5 Mice were dosed with 6Gy radiation and 5 mice with 15Gy.

Perfusion was quantified according to Equation 1 using mean signals from manually contoured regions-of-interest, highlighting the hippocampus, on each of the individual FAIR images for each animal with MIPAV software (National Institutes of Health, Bethesda, MD).

### Histological Analysis: Immunohistochemistry and Immunofluorescence

Mice were sacrificed according to instituitional guidelines and as previously described, using PFA perfusion fixation. Two hours prior to sacrificing mice Bromodeoxyuridine (BrDU) 50 mg/kg (Sigma) was injected intraperitoneally to highlight proliferation and 30 min prior to sacrifice Evans blue was injected 1 ml/kg of a 2% solution intravenously to highlight increasing permeability. Brains were removed and stored in 4% PFA for 24 hours. Brains were dehydrated in 30% sucrose before being cross-sectioned coronally at the site of the ICW and embedded in OCT freezing compound (Tissue-Tek) in a 1 cm^2^ mould, snap frozen in liquid nitrogen and store at −80°C. Frozen 5 µm sections were air dried at room temperature for 20 min. For immunohistochemical staining, endogenous peroxidase and biotin activities were blocked respectively using glucose oxidase and avidin/biotin blocking kits (Lab Vision). Serum blocking was carried out for 10 min using 5% serum derived from the secondary antibody source. Sections were incubated for one hour at room temperature with the primary antibodies B220 (Ebiosciences 1∶100), BrDU (Chemicon, 1∶200), CD11b (BD Pharmingen, 1∶50), CD11c (BD Pharmingen, 1∶50), CD3 (Abcam 1∶100), CD31 (BD Pharmingen, 1∶500), GFAP (Dako, 1∶4000), IBA1 (Wako, 1∶200), Ki67 (Dako 1∶50), MAC3 (BD Pharmingen, 1∶100), SMA (Abcam, 1∶1000). Biotinylated secondary (Vector labs) or fluorescent secondary (Invitrogen) to the primary antibodies were applied for one hour. Immunohistochemical stains underwent a 30 min horseradish peroxidase-conjugated ultrastreptavidin labeling (ID labs) treatment and color was developed using freshly prepared NovaRed solution (Vector labs), slides were lightly counterstained with hematoxylin, dehydrated in alcohols, cleared in xylene and mounted in Permount (Fisher). Immunofluorescent stained slides were mounted using a DAPI aqueous mount (Vector Labs) and were stored below 0°C. For the dual CD31:TUNEL immunohistochemical staining the primary antibody CD31 was followed with a biotinylated secondary for 30 min and horseradish peroxidase-conjugated ultrastreptavidin labeling reagent for 30 min. After washing well in TBS, color development was done with freshly prepared DAB solution. Sections were washed well in 3 changes of distilled water. For the second marker, TUNEL, slides were placed in cold Alcohol/Acetic acid mixture (2∶1) for 5 minutes and rinsed with PBS. Sections were then treated with buffer A for 5–10 min,and incubated in Biotin-nucleotide cocktail in a water bath at 37°C for 1 to 1.5 hr. After washing well in PBS Alkaline Phosphatase Strepavidin Reagent was applied for 30 minutes (Vector labs SA-5100). Further washes in PBS were carried out before color was developed with freshly prepared Vector Red solution (Vector Labs SK-5100).

### Semi-Quantification Analysis

Ten fields of view at 20× were blindly analyzed using Image J software. First RGB channels were split and converted to 8bit gray scale images. Background subtraction of 50 pixels equalized all channels. Threshold levels were adjusted to 45 pixels (min) to 225 pixels (max) in all images to mask the ‘particles’, upon applying the mask the images were converted to binary images for analysis. Particle analysis was completed based upon size restrictions of 8 pixel^2^–infinity leaving morphology unspecified due to the heterogeneous differentiation of the BMDCs, Z-stack images were analyzed as a block image to allow a semi-quantification of BMDCs/volume of tissue.

### Statistical Analysis

Experiments were carried out in triplicate. For multi-group comparison an ANOVA statistical test was performed, whilst for direct comparisons unpaired two-tailed Student's t-tests were carried out. We defined *significance at p<0.05. Data was analyzed using group mean, (n values appear on graphs) with error bars reported as standard deviation (*s.d.*).

All animal procedures were carried out according to animal user protocols approved ethically by the University Health Network Animal Care Committee under the guidelines of the Canadian Council on Animal Care..

## Supporting Information

Figure S1 **Further Characterization of Radiation Effect.** (**A**) Evans blue perfusion highlights area of increased leakiness in the right irradiated side of the brain, circled, when compared to the left non-irradiated side, Macroimaged. (**B**) Enlarged IBA1 immunofluorescence to further highlight the overlay with BMDCs in 50% of cases *(Green: IBA1, Red: BMDC, Blue: nuclei)*, 40× magnification.
(TIF)

Figure S2 **MRI analysis or Cerebral Blood Flow.** (**A**) Using FAIR MRI parameters images were uploaded into MiPav software and cerebral blood flow maps were generated. (**B**) Graphs demonstrating the CBF in both the sub-cortical and hippocampal regions of both the non-irradiated (L) hemisphere and irradiated (R) hemisphere, demonstrating there is no significant difference in flow either short term, day 1,3 post RT or long term, day 21 post RT.
(TIF)

Figure S3 **Imaging capabilities.** (**A**) Flattened out Z-stack image, showing the depth of resolution *(Green: BMDC, Blue: CD31)*, 10× magnification. White dashed box demonstrates the position of zoomed in images in (**B**). (**B**) Progressive photos through Z-stack to demonstrate the definition of cellular morphology available through the use of Z-stack imaging. It also demonstrates the closeness of the BMDCs to the vessel walls *(Green: BMDC, Blue: CD31)*, 10× magnification.
(TIF)

Figure S4 **Experimental Design and Layout.** Schemata identifying the use of mice along the experimental timeline highlighting the ability to re image and sac mice at each time point to corroborate imaging with histology.
(TIF)

Figure S5 **Bone Marrow Chimeric Engraftment.** (**A**) Up to 6 weeks post reconstitution extracted bone marrow can be examined and analyzed for both +ve (blue circles) and −ve (Green circles) fluorescence, to calculate +ve BM uptake as a % of total cell number. A high magnification image shows more clearly the cells defined as +ve and −ve, note that BM in this specimen was reconstituted with red fluorescent BM. (**B**) % BM uptake remains high at 72% even 6 weeks after the BM reconstitution with very little variation.
(TIF)

Figure S6 **Stereotactic Image Guided Radiation.** The in-house designed micro-irradiator allows for pinpoint accuracy of radiation delivery to the mice. (**A**) 360° Computer Tomography imaging is used to position the mouse, ensuring the iso-centre of radiation, red target, is in the centre of the right hemisphere where the ICW was generated. (**B**) An 8*11 mm collimator is used to ensure there is no leakage of radiation to further areas of the brain and further CT imaging can be used to position more accurately. (**C**) The radiation beam is administered from a gantry, black arrowhead, which rotates 360° allowing the radiation to be administered from any position. The mouse platform remains static in the centre of the radiator, white arrowhead, whilst radiation is administered A-P, from the top and P-A, from the bottom. This prevents a gradient of radiation through the tissue as it travels further from the source.
(TIF)

Video S1 **Time Lapse Imaging of BMDCs.** Time Lapse images capturing a 150 msec time window demonstrates the ability of the imaging to track dynamically the movement of BMDCs and reconstituted blood through the vessels, (*Green: BMDCs, Blue: CD31*) 10× magnification.
(MOV)

Video S2 **Z-stack imaging of BMDCs.** Z-Stack images capturing a 150 µm depth section of the window every 10 µm demonstrates the ability of the imaging to enhance the three dimensional morphology of BMDCs and their relationship to the vessels, (*Green: BMDCs, Blue: CD31*) 10× magnification.
(MOV)
