# Supplementary figures and images for: High-Resolution In-Vivo Analysis of Normal Brain Response to Cranial Irradiation
Source: PLoS One. 2012 Jun 4;7(6):e38366. doi: 10.1371/journal.pone.0038366 (PMC3366930; doi:10.1371/journal.pone.0038366)

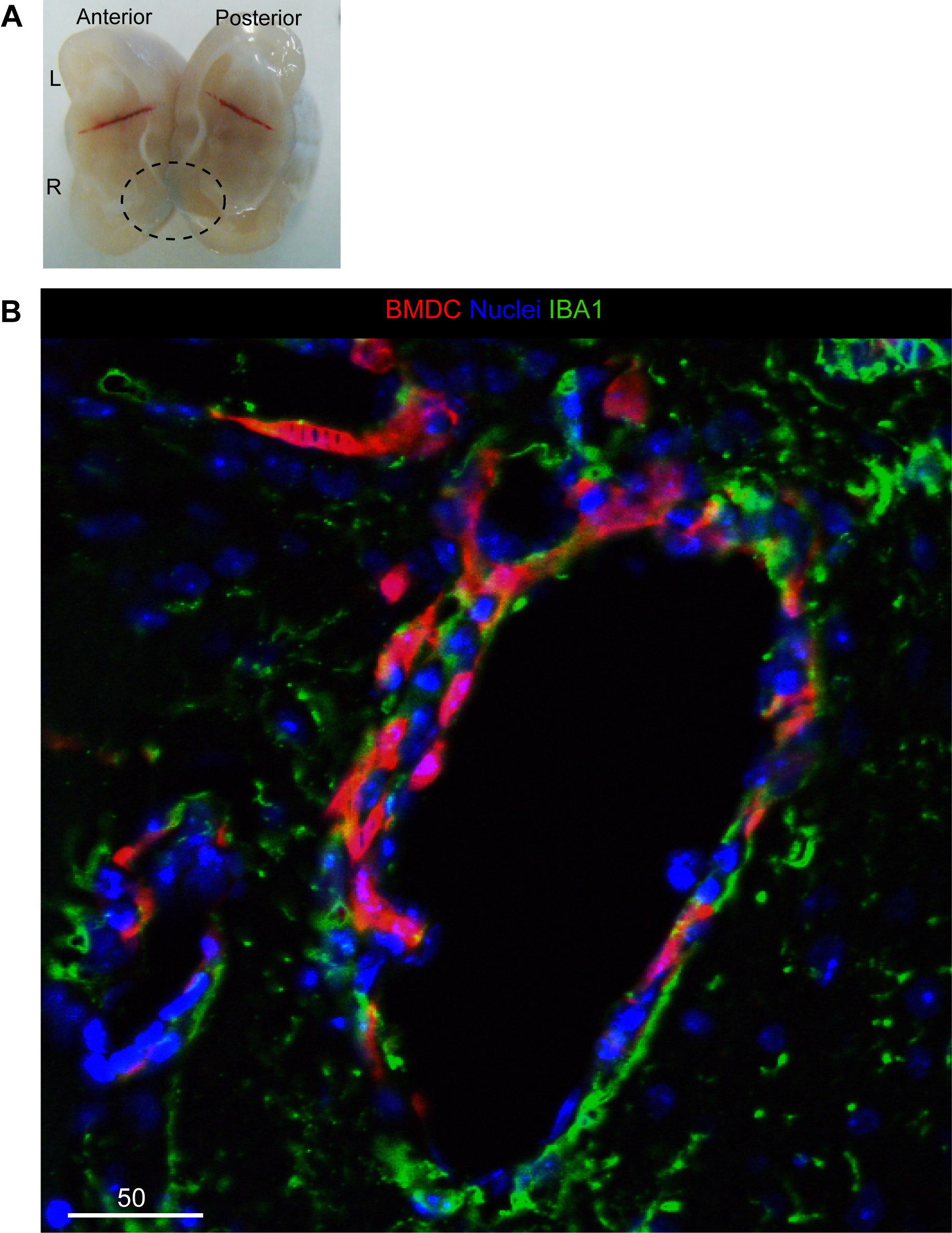

Supplement: Figure S1 — Further Characterization of Radiation Effect. (A) Evans blue perfusion highlights area of increased leakiness in the right irradiated side of the brain, circled, when compared to the left non-irradiated side, Macroimaged. (B) Enlarged IBA1 immunofluorescence to further highlight the overlay with BMDCs in 50% of cases (Green: IBA1, Red: BMDC, Blue: nuclei), 40× magnification. (TIF) [file pone.0038366.s001.tif]

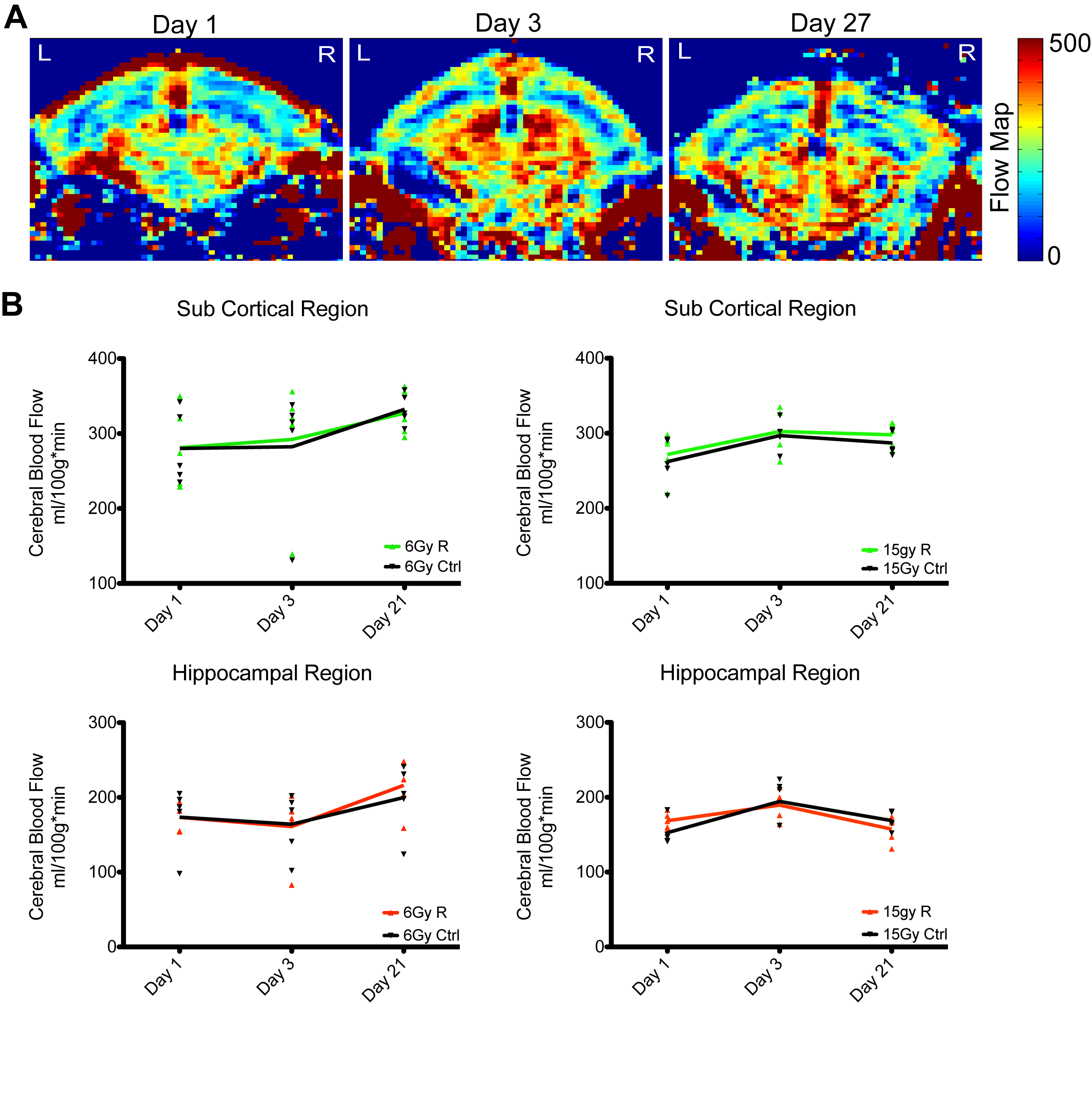

Supplement: Figure S2 — MRI analysis or Cerebral Blood Flow. (A) Using FAIR MRI parameters images were uploaded into MiPav software and cerebral blood flow maps were generated. (B) Graphs demonstrating the CBF in both the sub-cortical and hippocampal regions of both the non-irradiated (L) hemisphere and irradiated (R) hemisphere, demonstrating there is no significant difference in flow either short term, day 1,3 post RT or long term, day 21 post RT. (TIF) [file pone.0038366.s002.tif]

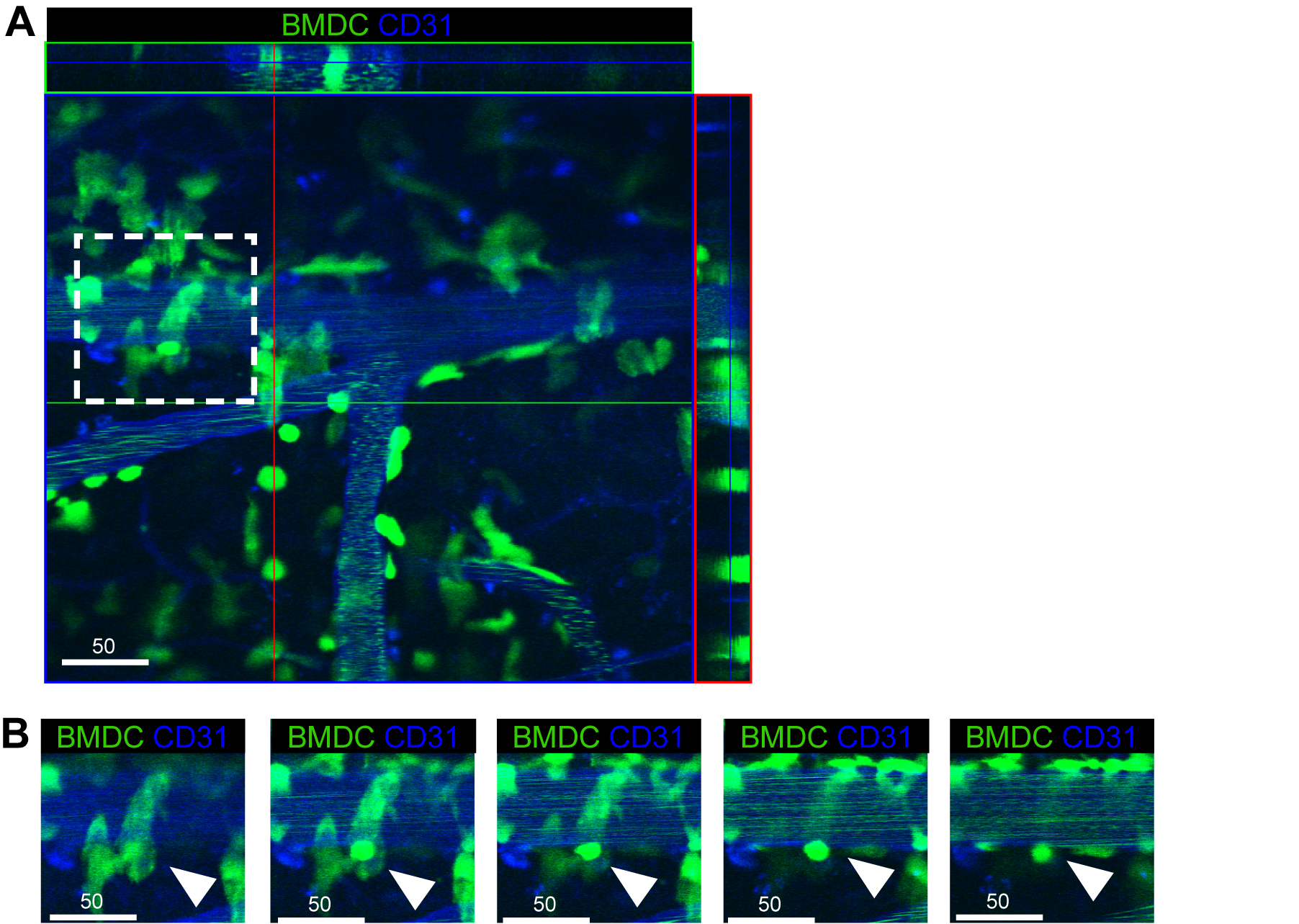

Supplement: Figure S3 — Imaging capabilities. (A) Flattened out Z-stack image, showing the depth of resolution (Green: BMDC, Blue: CD31), 10× magnification. White dashed box demonstrates the position of zoomed in images in (B). (B) Progressive photos through Z-stack to demonstrate the definition of cellular morphology available through the use of Z-stack imaging. It also demonstrates the closeness of the BMDCs to the vessel walls (Green: BMDC, Blue: CD31), 10× magnification. (TIF) [file pone.0038366.s003.tif]

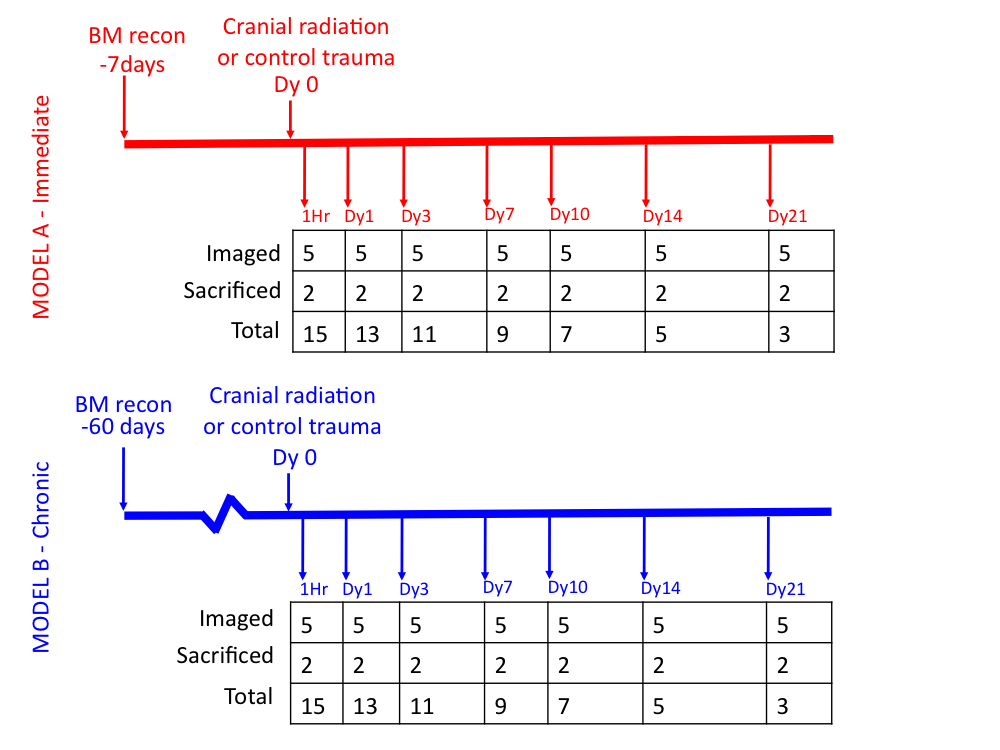

Supplement: Figure S4 — Experimental Design and Layout. Schemata identifying the use of mice along the experimental timeline highlighting the ability to re image and sac mice at each time point to corroborate imaging with histology. (TIF) [file pone.0038366.s004.tif]

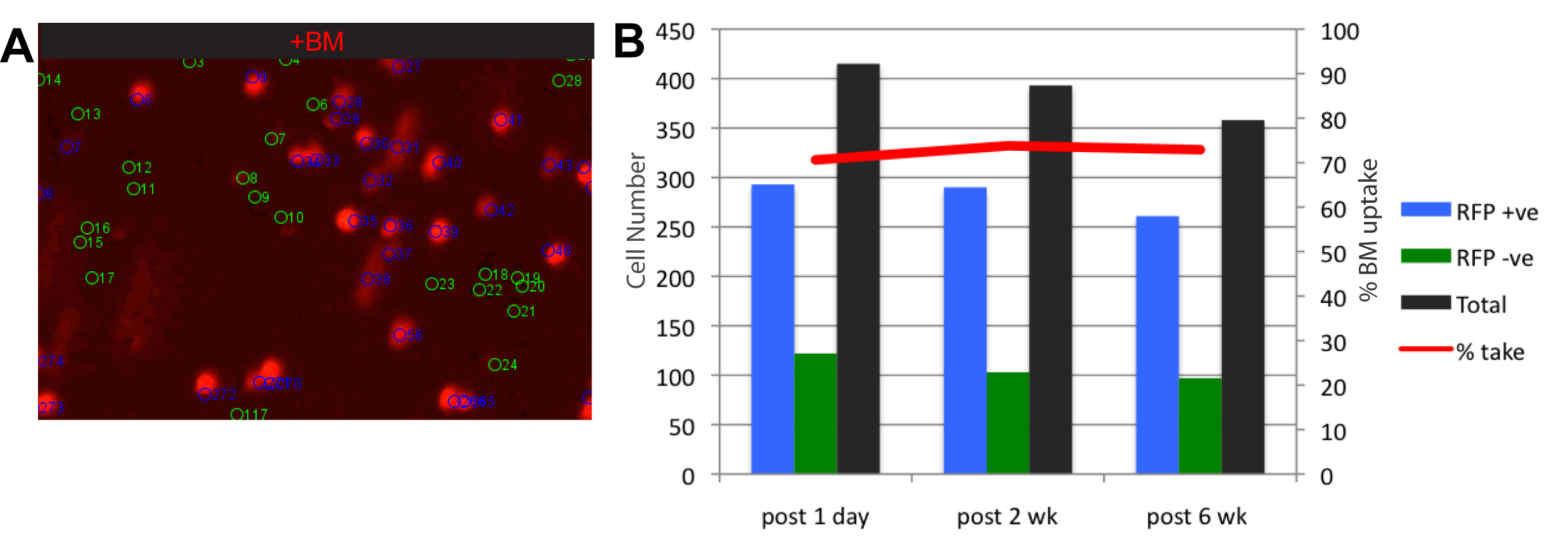

Supplement: Figure S5 — Bone Marrow Chimeric Engraftment. (A) Up to 6 weeks post reconstitution extracted bone marrow can be examined and analyzed for both +ve (blue circles) and −ve (Green circles) fluorescence, to calculate +ve BM uptake as a % of total cell number. A high magnification image shows more clearly the cells defined as +ve and −ve, note that BM in this specimen was reconstituted with red fluorescent BM. (B) % BM uptake remains high at 72% even 6 weeks after the BM reconstitution with very little variation. (TIF) [file pone.0038366.s005.tif]

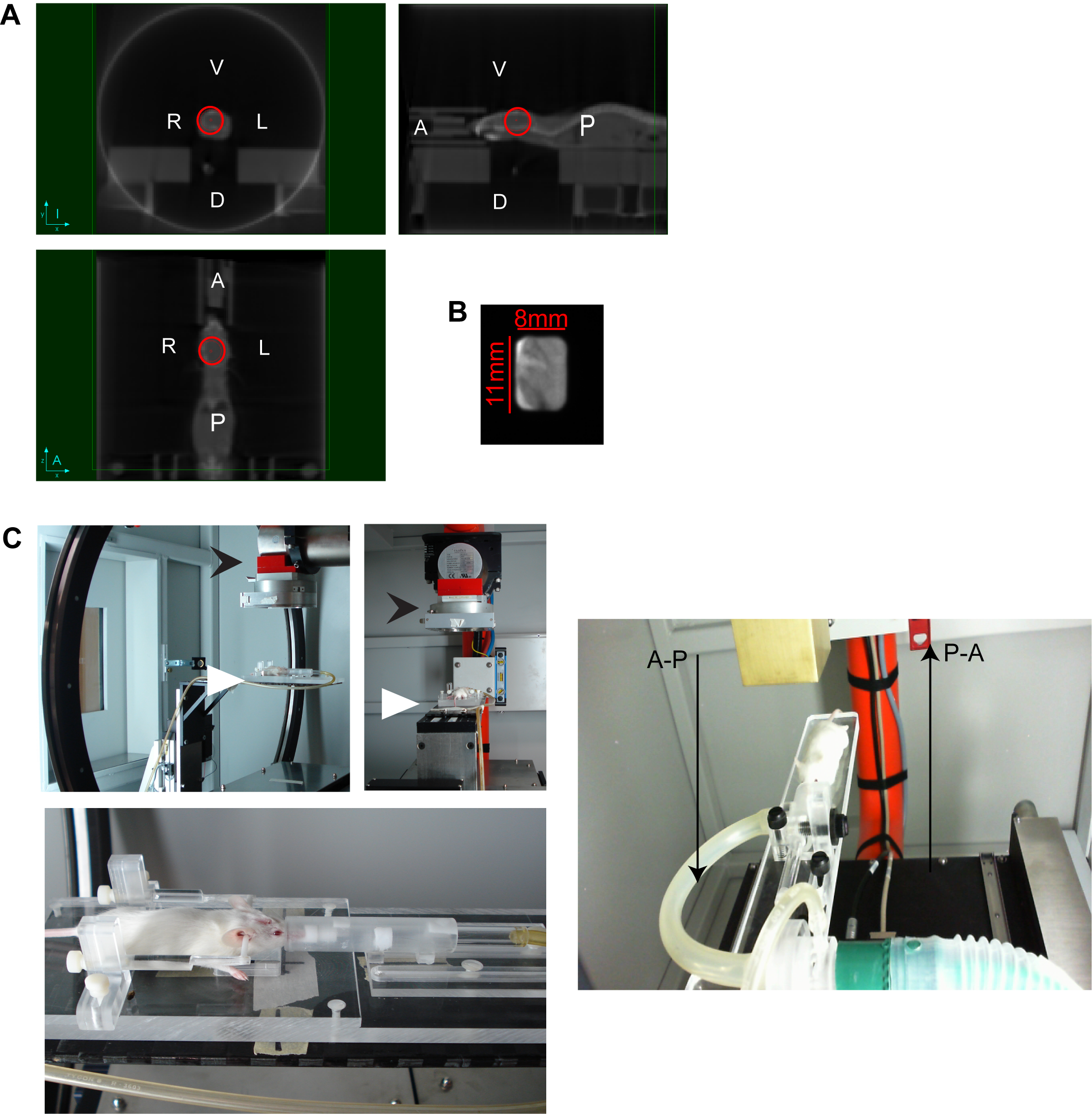

Supplement: Figure S6 — Stereotactic Image Guided Radiation. The in-house designed micro-irradiator allows for pinpoint accuracy of radiation delivery to the mice. (A) 360° Computer Tomography imaging is used to position the mouse, ensuring the iso-centre of radiation, red target, is in the centre of the right hemisphere where the ICW was generated. (B) An 8*11 mm collimator is used to ensure there is no leakage of radiation to further areas of the brain and further CT imaging can be used to position more accurately. (C) The radiation beam is administered from a gantry, black arrowhead, which rotates 360° allowing the radiation to be administered from any position. The mouse platform remains static in the centre of the radiator, white arrowhead, whilst radiation is administered A-P, from the top and P-A, from the bottom. This prevents a gradient of radiation through the tissue as it travels further from the source. (TIF) [file pone.0038366.s006.tif]
